# Dual-colour (near-infrared/visible) emitting annexin V for fluorescence imaging of tumour cell apoptosis *in vitro* and *in vivo*†

**DOI:** 10.1039/d0ra06495e

**Published:** 2020-10-16

**Authors:** Setsuko Tsuboi, Takashi Jin

**Affiliations:** RIKEN Center for Biosystems Dynamics Research (BDR) RIKEN Furuedai 6-2-3 Suita Osaka 565-0874 Japan tjin@riken.jp; Graduate School of Frontier Biosciences, Osaka University Yamada-oka 1-3 Suita Osaka 565-0871 Japan

## Abstract

Indocyanine green (ICG) labelled recombinant annexin V proteins (ICG–EGFP–Annexin V and ICG–mPlum–Annexin V) were synthesized for dual-colour fluorescence imaging of tumour cell apoptosis *in vitro* and *in vivo*. The ICG-labelled fluorescent annexin V proteins showed dual (near-infrared and visible) fluorescence emissions with binding ability to phosphatidylserines on the plasma membranes of apoptotic cells. Although several types of fluorescence labelled annexin V (*e.g.* FITC–annexin V, Cy3- and Cy5-annexin V) have been reported, there are no dual-colour (near-infrared/visible) emitting apoptosis-detection probes which can be used *in vitro* and *in vivo*. In this paper, the utilities of the dual-colour fluorescent annexin V are demonstrated for *in vitro* and *in vivo* fluorescence imaging of the apoptosis of human breast tumour cells induced by an antibody–drug conjugate, Kadcyla. The results suggest that the present annexin V probes will be useful to visualize the action of anti-cancer drugs against tumours both at the cellular and whole-body level.

## Abstract 2

Indocyanine green labeled recombinant annexin V probes (ICG–EGFP–Annexin V and ICG–mPlum–Annexin V) were synthesized for near-infrared and visible fluorescence imaging of tumor cell apoptosis both *in vitro* and *in vivo.*

## Graphical abstract

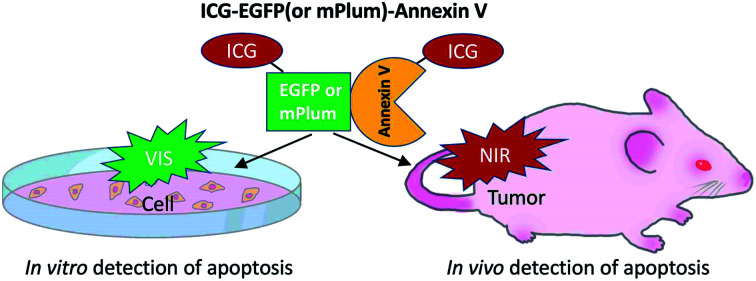

## Introduction

Recent development of antibody–drug conjugates (ADCs) enables specific binding of anti-cancer drugs to tumour cells for apoptosis-based cancer therapy.^1^*In vivo* visualization of tumour apoptosis induced by ADCs is crucial for the evaluation of action of the drugs in cancer therapy and for the development of cancer therapeutics.^2^ For *in vivo* imaging of ADC-induced apoptosis, we can use several imaging modalities such as single photon emission computed tomography (SPECT), positron emission tomography (PET), and magnetic resonance imaging (MRI).^3–7^ SPECT and PET need to use radioactive tracers for *in vivo* imaging, where their spatial resolution is not enough to visualize apoptotic cells.^5,8^ Although MRI enables *in vivo* three-dimensional imaging with a high spatial resolution (0.1–1 mm), the detection sensitivity of MRI is relatively low.^4^ An alternative of the imaging modality for *in vivo* detection of apoptotic cells is optical imaging.^4,8^ Compared to SPECT, PET and MRI, optical imaging has a high tempo-spatial resolution (ms μm^−1^).^8^ Herein, we present non-invasive optical imaging of ADC-induced apoptosis of breast tumour cells *in vitro* and *in vivo* using near-infrared (NIR) and visible (VIS) emitting fluorescent annexin V.

Apoptosis can be induced in cancer cells through intrinsic and extrinsic pathways without inducing an inflammatory response.^9^ During the early process of apoptosis, morphological changes in the plasma membrane induce the asymmetrical distribution of phosphatidylserine (PS) on the outer monolayer of the membrane.^10^ Annexin V, an endogenous protein with binding ability to PS in the presence of Ca^2+^ ions, is widely used as an optical probe for the detection of apoptosis.^11^ To date, a variety of apoptosis probes based on annexin V modified with fluorescence, luminescence, magnetic resonance contrast, and radioisotopes have been developed.^12–30^ Among these probes, fluorescein conjugated annexin V (FITC–Annexin V)^9^ is a most popular probe for the detection of apoptotic cells. FITC–Annexin V has been widely used as a fluorescent probe for the apoptosis detection by fluorescence microscopy and fluorescence-activated cell sorting methods.^31^ However, FITC–Annexin V is not suitable for *in vivo* imaging of apoptotic cells due to its VIS fluorescence, which is strongly absorbed and scattered by tissues and organs.

**Scheme 1:**
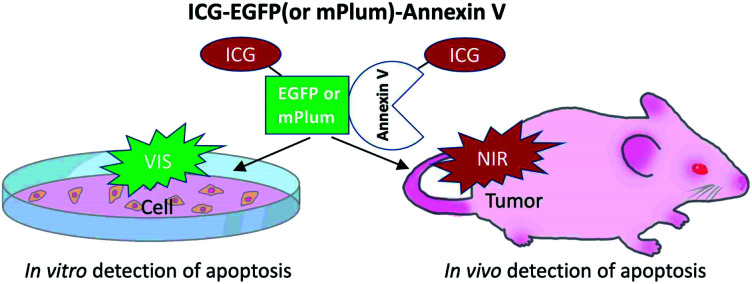
NIR/VIS emitting dual-colour probes (ICG–EGFP–Annexin V and ICG–mPlum–Annexin V) for fluorescence imaging of tumour cell apoptosis *in vitro* and *in vivo*.

Although a few types of NIR-fluorescence labelled annexin V (*e.g.* Cy5-annexin V^29,30^ and 800CW-annexin V^27^) have been reported, there are no NIR and VIS emitting dual-colour probes which can be used for the detection of apoptosis both at the cellular (*in vitro*) and whole-body level (*in vivo*). In this work, we developed indocyanine green (ICG) labelled recombinant fluorescent annexin V^32^ probes (ICG–EGFP^33^–Annexin V and ICG–mPlum^34^–Annexin V, Scheme 1) that enable both *in vitro* and *in vivo* fluorescence imaging of breast-tumour cell apoptosis induced by an ADC, trastuzumab emtansine^35^ (T-DM1, Kadcyla). Kadcyla is a conjugate between a humanized monoclonal anti-HER2 (human epidermal growth factor receptor 2)^36^ antibody and maytansinoid (DM1), a highly potent microtubule polymerization inhibitor.^35^ Kadcyla acts as an anti-cancer drug against HER2 positive cancer.^37^ By using the dual-colour (NIR/VIS) emitting annexin V probes, we achieved *in vitro* and *in vivo* imaging of apoptosis of HER2-positive breast tumour cells (KPL-4) treated with Kadcyla. Our findings suggest that the present dual-colour (NIR/VIS) emitting annexin V can be used for the evaluation of the action of ADCs against cancerous tumours both at the cellular and whole-body level.

## Results and discussion

### Probe design and characterization

**Fig. 1:**
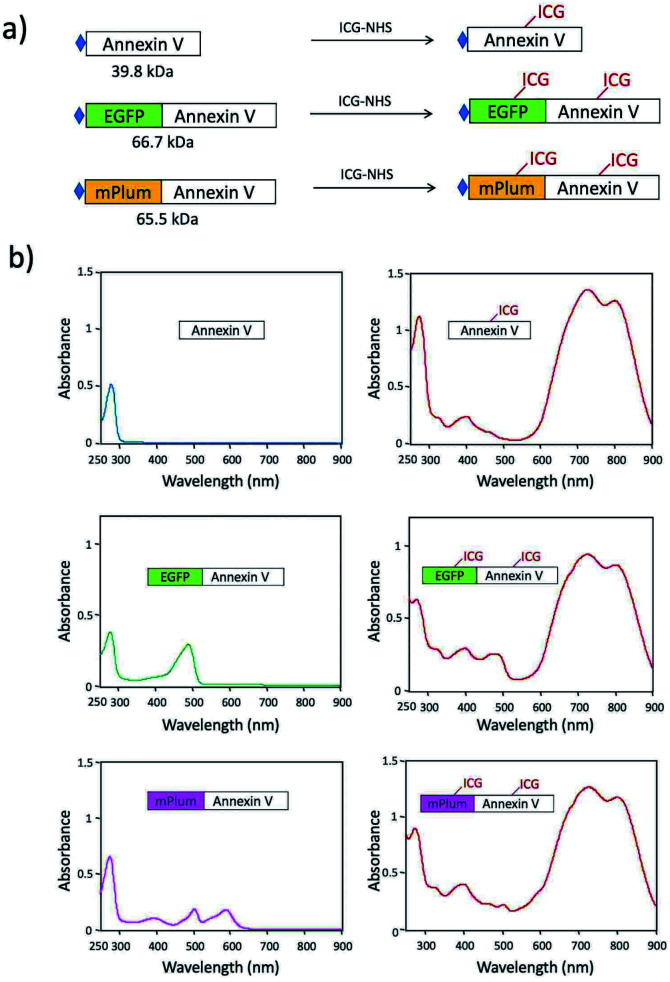
(a) Fluorescence labelling of Annexin V, EGFP–Annexin V, and mPlum–Annexin V with ICG-NHS. The symbol of 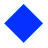 shows a histidine tag. (b) Absorption spectra of Annexin V, EGFP–Annexin V, and mPlum–Annexin V in phosphate buffered saline (PBS) before and after labelling with ICG-NHS.

For *in vivo* optical imaging of apoptotic cells, NIR probes emitting over 700 nm are desirable. VIS fluorescence (400 to 700 nm) is strongly absorbed and scattered by intrinsic chromophores and organelles in cells. In contrast, NIR fluorescence shows higher permeability and lower scattering in living tissues, leading to clearer deep-tissue images. To prepare NIR apoptosis-imaging probes, we labelled recombinant annexin V proteins with ICG (Fig. 1a, S1 and S2†). Annexin V is a 39.8 kDa molecule with 22 lysine residues, and it is easily labelled with *N*-succinimidyl ester of ICG (ICG-NHS). The molecular weight of EGFP–Annexin V and mPlum–Annexin V is 66.7 kDa and 65.5 kDa, where EGFP and mPlum have 19 and 20 lysine residues, respectively. EGFP and mPlum can also be labelled with ICG-NHS ester (Fig. S3†). From the absorption spectra of ICG-labelled recombinant proteins (Fig. 1b), the number of ICG molecule binding to one recombinant protein molecule was estimated to be 3, 4, and 5 for ICG–Annexin V, ICG–EGFP–Annexin V and ICG–mPlum–Annexin V, respectively.

**Fig. 2:**
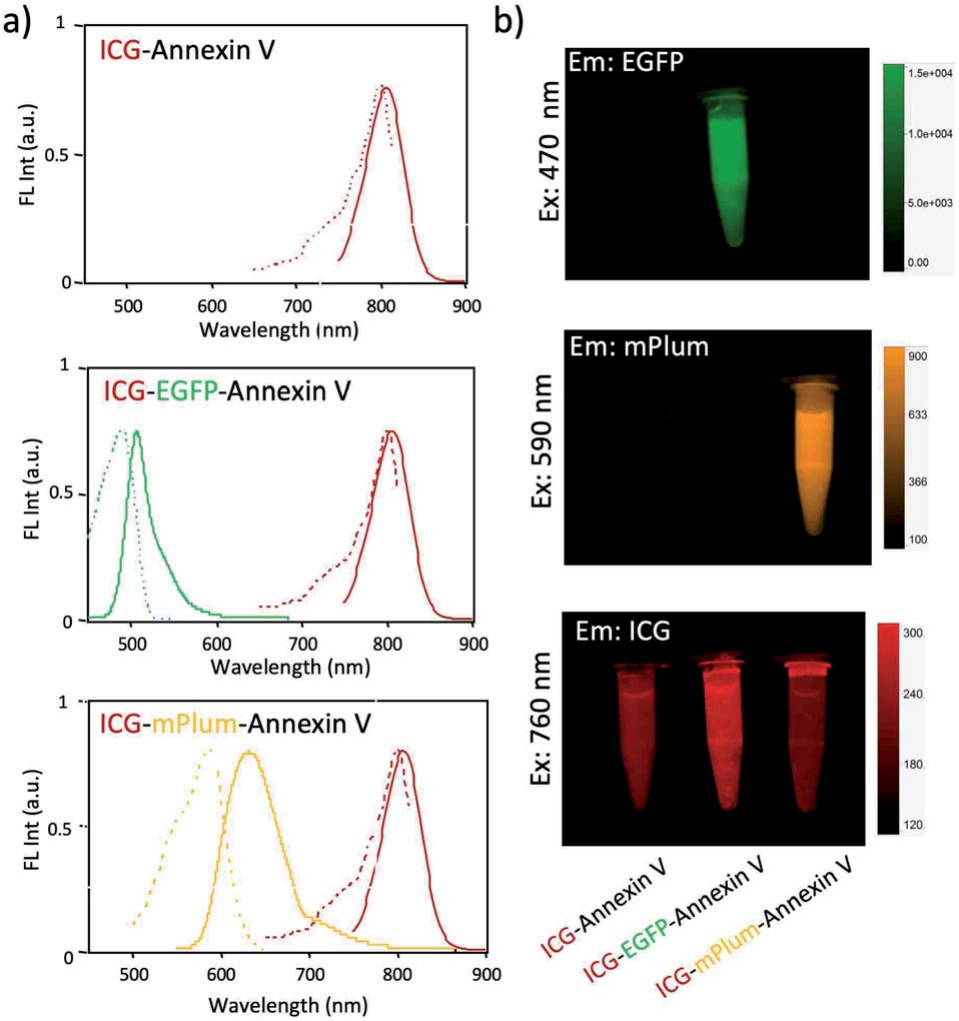
(a) Fluorescence spectra of ICG–Annexin V, ICG–EGFP–Annexin V, and ICG–mPlum–Annexin V in PBS. Solid and broken lines show their emission and excitation spectra, respectively. (b) Pseudo-coloured fluorescence images of ICG–Annexin V, ICG–EGFP–Annexin V, and ICG–mPlum–Annexin V in PBS. The fluorescence images of EGFP (em: 515 ± 20 nm), mPlum (em: 670 ± 20 nm), and ICG (em: 830 ± 20 nm) were taken by excitation at 470, 590, and 760 nm, respectively.

ICG–Annexin V shows an emission peak at 820 nm, while ICG–EGFP–Annexin V and ICG–mPlum–Annexin V show NIR/VIS dual-emission peaks resulting from ICG and fluorescent proteins (Fig. 2a). ICG–EGFP–Annexin V has emission maxima at 515 nm (for EGFP) and 820 nm (for ICG), where the quantum yield of EGFP and ICG emission was estimated to be 35% and *ca.*1% (Table S1†), respectively. ICG–mPlum–Annexin V has emission maxima at 640 nm and 820 nm, where the quantum yield of mPlum and ICG emission was 7% and *ca.*1% (Table S1†), respectively. To visualize the NIR/VIS dual-emission nature of ICG–EGFP–Annexin V and ICG–mPlum–Annexin V, we measured their fluorescence images by excitation at 470 nm for EGFP, 590 nm for mPlum, and 760 nm for ICG. ICG–EGFP–Annexin V and ICG–mPlum–Annexin V show green/NIR dual fluorescence and far-red/NIR dual fluorescence, respectively (Fig. 2b). This NIR and VIS dual emission property suggests that ICG–EGFP–Annexin V and ICG–mPlum–Annexin V can be used for the VIS and NIR fluorescence imaging of apoptotic cells both *in vitro* and *in vivo*.

**Fig. 3:**
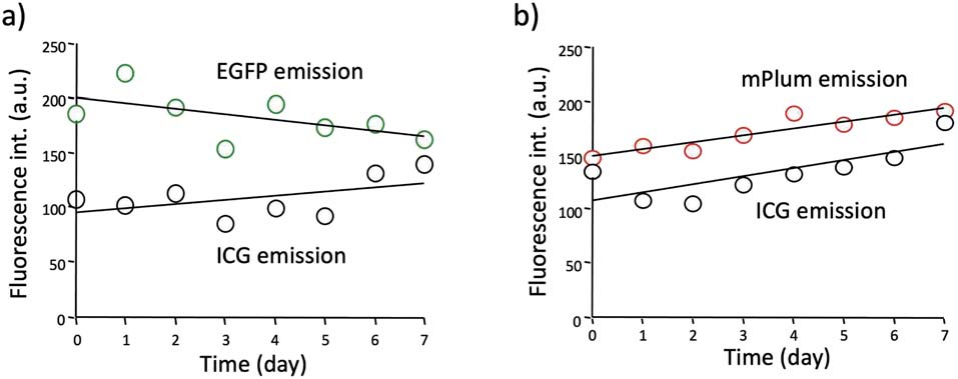
Time course of fluorescence intensities of ICG, EGFP and mPlum emissions in (a) ICG–EGFP–Annexin V and (b) ICG–mPlum–Annexin V in PBS. Fluorescence intensities are detected at 515 nm for EGFP, 670 nm for mPlum, and 830 nm for ICG. The concentration of probe was 15 μM.

Although ICG is a widely used NIR dye for *in vivo* fluorescence imaging, the drawback of ICG is low photo- and chemical-stabilities in physiological buffer solutions. For instance, ICG degrades in PBS resulting in a loss of absorption and fluorescence because of the formation of reduced non-fluorescence species.^38^ To check the practical usefulness of ICG–EGFP–Annexin V and ICG–mPlum–Annexin V, we examined their photostability in PBS. The time course of fluorescence intensity of ICG–EGFP–Annexin V and ICG–mPlum–Annexin V in PBS showed that the fluorescence emissions of ICG as well as EGFP and mPlum were not drastically changed for one week (Fig. 3). In addition, we observed that the irradiation of excitation lights (3 mW cm^−2^) to the aqueous solutions of ICG–EGFP–Annexin V and ICG–mPlum–Annexin V did not result in significant photo-bleaching (Fig. S4†).

### Fluorescence imaging of apoptotic breast tumour cells *in vitro*

**Fig. 4:**
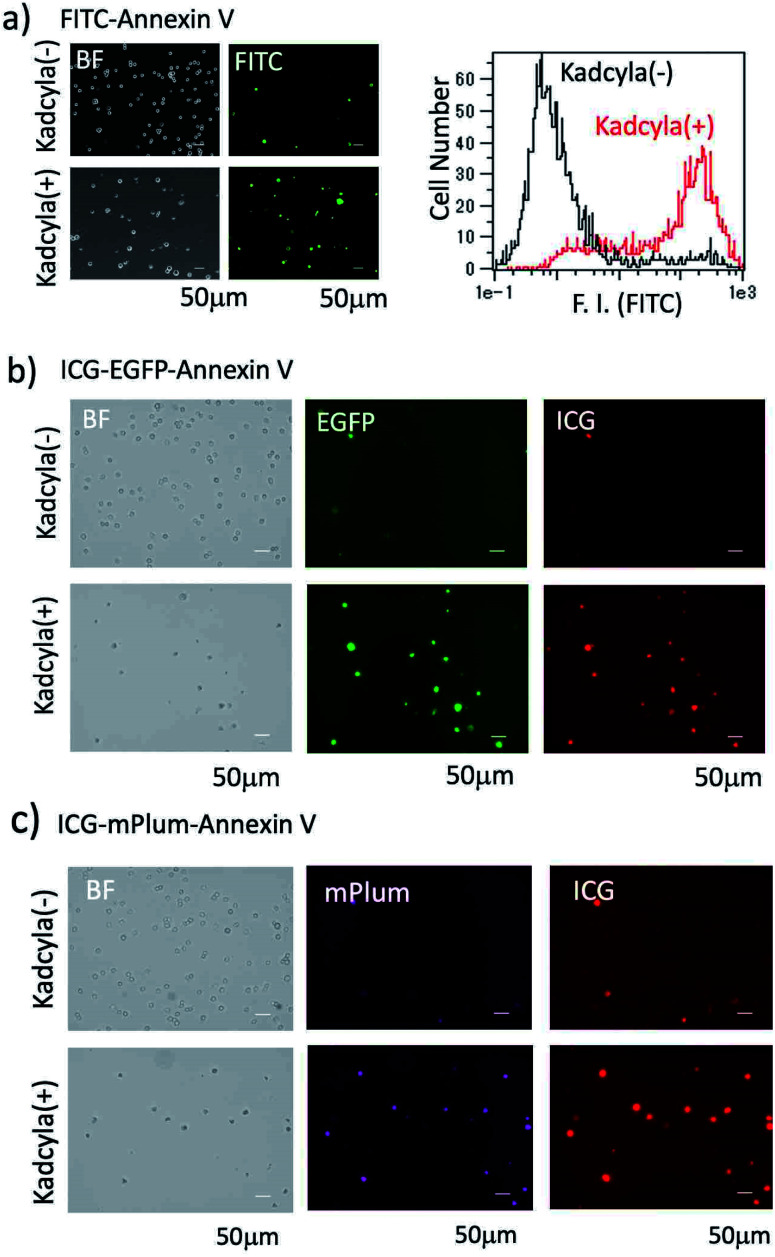
Bright field (BF) and fluorescence images of KPL-4 cells treated with and without Kadcyla (10 nM). Cellular imaging and flow cytometric analysis were performed three days after the treatment of Kadcyla. (a) KPL-4 cells were stained with FITC–Annexin V. Green colour shows the emission from FITC. The right graph shows the flow cytometric analysis of KPL-4 cells treated with and without Kadcyla. (b) KPL-4 cells were stained with ICG–EGFP–Annexin V. The emissions of EGFP (green) and ICG (red) were detected at 525 ± 25 nm (ex: 470 ± 20 nm) and 832 ± 19 nm (ex: 769 ± 20 nm), respectively. (c) KPL-4 cells were stained with ICG–mPlum–Annexin V. The emissions of mPlum (magenta) and ICG (red) were detected at > 590 nm (ex: 560 ± 20 nm) and 832 ± 19 nm (ex: 769 ± 20 nm), respectively.

For the fluorescence imaging of tumour cell apoptosis, we used a human breast tumour cell line (KPL-4),^39^ which overexpresses HER2 on its cell surface. To observe the apoptosis of KPL-4 cells, we first used a widely used FITC–Annexin V as an apoptosis-detection probe. Apoptosis of KPL4 cells was induced by Kadcyla. After the treatment of KPL-4 cells with Kadcyla for three days, fluorescence imaging of KPL-4 cells was performed by using FITC–Annexin V (Fig. 4a). The KPL-4 cells treated with Kadcyla showed the significant emission of fluorescein (green emission) from the plasma membrane of KPL-4 cells, indicating that the apoptosis of KPL-4 cells was induced by Kadcyla (Fig. 4a and S5†). In contrast, the control KPL-4 cells (with no Kadcyla) showed only a weak fluorescein emission from the cell membrane Fig. 4a. Flow cytometric analysis showed that the apoptosis of KPL-4 cells was induced by a factor of 76% by treated with 10 nM of Kadcyla for 72 h (a right graph in Fig. 4a).

We next checked whether the apoptosis of KPL-4 cells can be detected by the fluorescence of ICG–EGFP–Annexin V and ICG–mPlum–Annexin V. In this case, we performed VIS and NIR dual-colour fluorescence imaging of the apoptosis of KPL-4 cells. Fluorescence images of KPL-4 cells by incubated with ICG–EGFP–Annexin V and ICG–mPlum–Annexin V showed the VIS and NIR intense emissions from the cells treated with Kadcyla, but not from the cells treated with no Kadcyla (Fig. 4b and c). This result shows that ICG–EGFP–Annexin V and ICG–mPlum–Annexin V can be used as VIS/NIR dual-colour fluorescent probes for the detection of apoptosis *in vitro*. Blocking experiments using unlabelled Annexin V supported the practical usefulness of ICG–EGFP–Annexin V and ICG–mPlum–Annexin V as fluorescent probes for the detection of apoptotic cells (Fig. S6†).

### Breast-tumour bearing mice

**Fig. 5:**
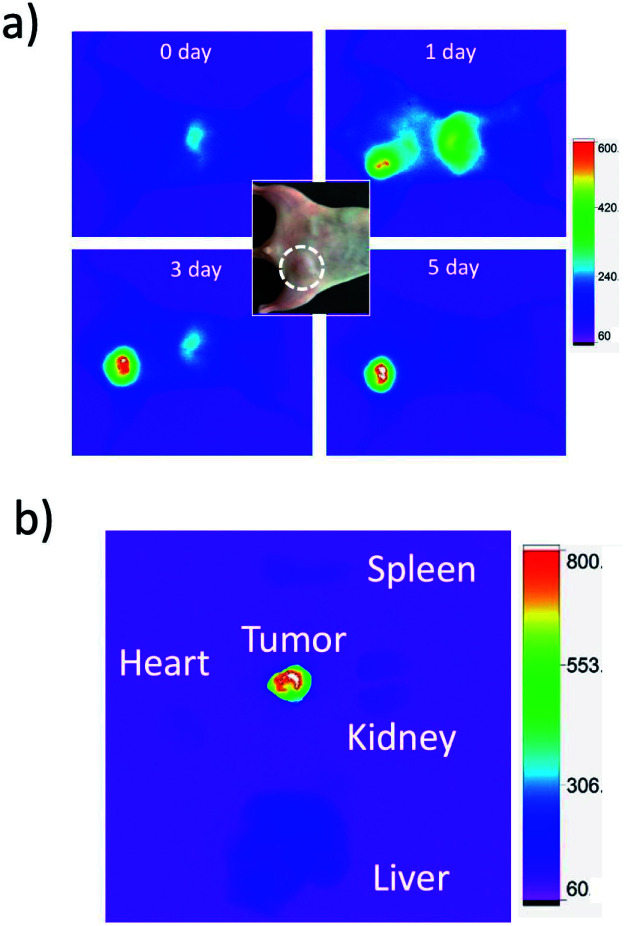
NIR fluorescence images of a breast-tumour bearing mouse, where an aqueous solution (200 μL) of ICG-labelled Kadcyla (1 mg mL^−1^) was intravenously injected *via* a tail vein of the mouse. A dotted circle in the bright field image of a mouse shows the position of a tumour. (a) NIR fluorescence images of the mouse were taken 0, 1, 3, and 5 days after the injection of ICG labelled Kadcyla. (b) *Ex vivo* image shows NIR fluorescence emission from an isolated breast tumour and organs. NIR fluorescence (em: 830 ± 20 nm) was observed by excitation at 760 nm. Exposure time was 30 s.

For *in vivo* imaging of tumour apoptosis, we used breast-tumour bearing mice. Breast-tumour bearing mice were prepared by implanting of KPL-4 cells to nude mice.^39*c*^ Tumour apoptosis was induced by an intravenous injection of Kadcyla to the nude mouse. First, we checked whether Kadcyla can accumulate to a HER2-positive breast tumour in the nude mouse. For *in vivo* fluorescence imaging of Kadcyla, we labelled Kadcyla with ICG, and ICG-labelled Kadcyla was intravenously injected to the mouse *via* its tail vein. Immediately after the injection of ICG-labelled Kadcyla, fluorescence emission of ICG was observed from the liver (Fig. 5a). Three days after the injection, intense fluorescence emission of ICG was observed from the tumour, indicating the accumulation of Kadcyla to the HER2-positive breast tumour. *Ex vivo* fluorescence imaging of a breast tumour and organs clearly showed the specific accumulation of Kadcyla to the breast tumour (Fig. 5b).

### Fluorescence imaging of apoptotic breast tumour cells *in vivo*

**Fig. 6:**
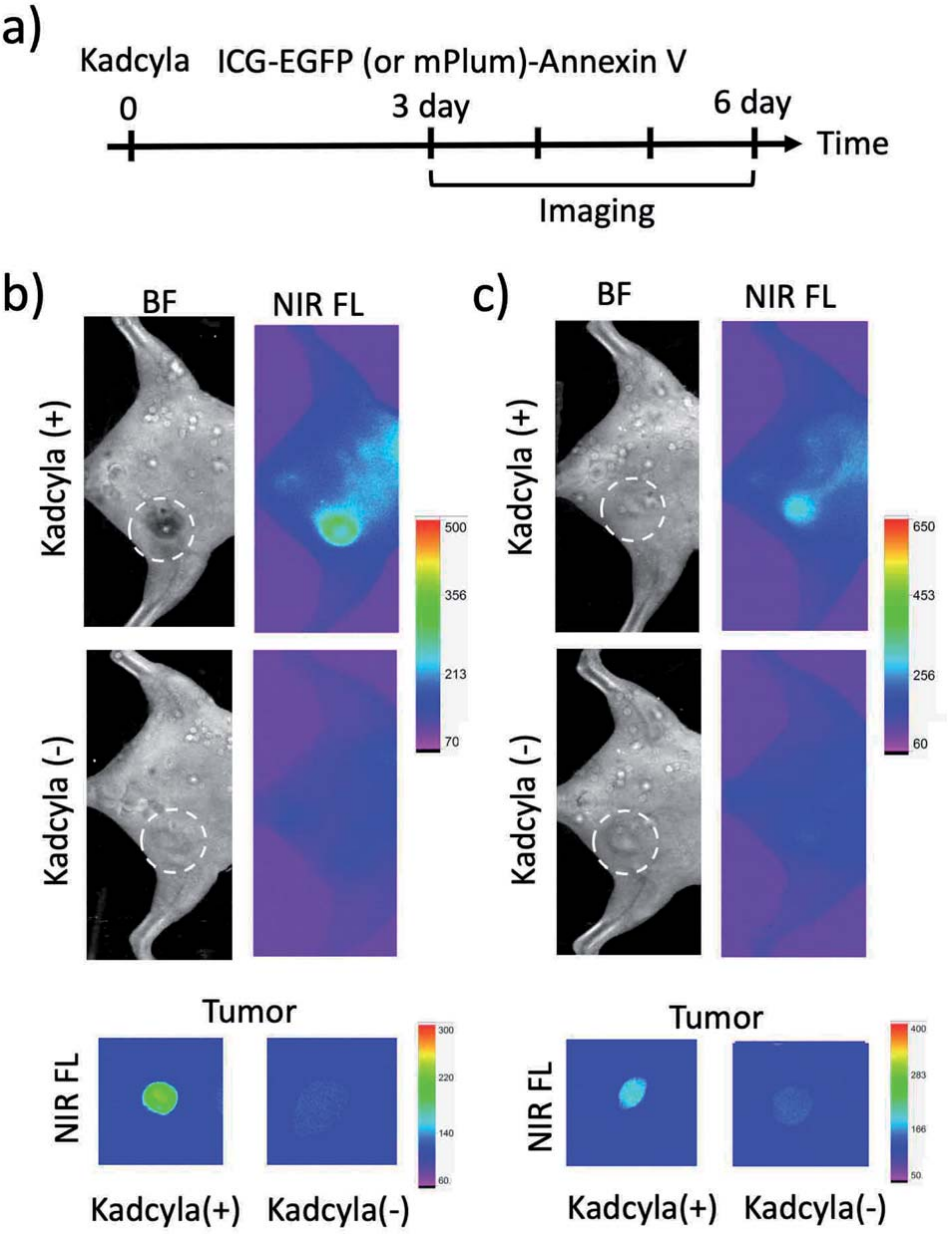
(a) Time course of experimental procedure for Kadcyla, ICG–EGFP (or mPlum)–Annexin V administration and fluorescence imaging. Kadcyla (200 μL, 1 mg mL^−1^) was intravenously injected *via* a tail vein of breast tumour-bearing mice. ICG–EGFP (or Plum)–Annexin V was injected to the mice three days after the injection of Kadcyla. (b) Bright filed and NIR fluorescence (ICG–EGFP–Annexin V) images of a mouse treated with and without Kadcyla. (c) Bright filed and NIR fluorescence (ICG–mPlum–Annexin V) images of a mouse treated with and without Kadcyla. Dotted circles show the positions of breast tumours in the mice. *Ex vivo* images show NIR fluorescence emissions from isolated breast tumours. NIR fluorescence (em: 830 ± 20 nm) was observed by excitation at 760 nm. Exposure time was 30 s.

To image tumour apoptosis, we injected ICG–EGFP–Annexin V and ICG–mPlum–Annexin V to the breast tumour-bearing mice treated with Kadcyla for three days (Fig. 6a). Fluorescence imaging of the mice was started three days after the injection of Kadcyla (Fig. 6a). Fluorescence emission of ICG was observed from a breast tumour one day after the injection of ICG–EGFP–Annexin V (Fig. S7†). After three days after the injection of the ICG–EGFP (or mPlum)–Annexin V, significant ICG fluorescence from the breast tumour was clearly observed (Kadcyla (+) in Fig. 6b and c), indicating the accumulation of the Annexin V probes to the tumour. In the VIS region, EGFP or mPlum emission from the tumour was not clearly observed due to the strong emission of tissue autofluorescence (Fig. S8†). ICG–Annexin V also accumulated to the breast tumour treated with Kadcyla (Fig. S9†). For the control mice treated with no Kadcyla, we observed only weak fluorescence emissions resulting from the accumulation of ICG–EGFP–Annexin V and ICG–mPlum–Annexin V to the tumour (Kadcyla (−) in Fig. 6b and c). The emission intensity ratio between the breast tumours treated with and without Kadcyla was 6.3 for an ICG–EGFP–Annexin V-injected mouse and 2.4 for an ICG–mPlum–Annexin V-injected mouse (lower images in Fig. 6b and c).

To confirm the specific accumulation of ICG–EGFP–Annexin V and ICG–mPlum–Annexin V to the apoptotic tumour, we performed a control experiment using ICG labelled EGFP (ICG–EGFP) and ICG-labelled mPlum (ICG–mPlum), which have no annexin V moieties.

When ICG–EGFP and ICG–mPlum with no annexin V moieties were injected to breast-tumour bearing mice treated with Kadcyla, significant fluorescence emissions were not observed from the tumours (Fig. S10†). The control experiment clearly showed that ICG–EGFP–Annexin V and ICG–mPlum–Annexin V accumulated to the tumour by binding of its annexin V moiety to PS molecules on the plasma membrane of apoptotic tumour cells.

## Conclusion

In this paper, we present ICG labelled recombinant annexin V probes (ICG–EGFP–Annexin V and ICG–mPlum–Annexin V) for NIR and VIS fluorescence imaging of tumour cell apoptosis. The objective of this work is to develop new fluorescent annexin V probes which can be used for the detection of apoptosis both at the cellular and whole-body level. Although several types of fluorescent annexin V have been reported, there are no fluorescent annexin V probes which can be used for the optical detection of apoptotic cells both *in vitro* and *in vivo.* By using the present annexin V probes, we demonstrate the capability of VIS and NIR dual-colour imaging for the detection of apoptosis. Deregulation in apoptosis induces numerous diseases such as cancer, cardiovascular, and neurodegenerative diseases. We believe that the present VIS/NIR dual-colour emitting annexin V probes will greatly contribute to the study of apoptosis-related diseases at the whole-body level as well as the cellular level.

## Experimental

### Materials and instruments

*N*-Succinimidyl ester derivative of ICG (ICG-NHS) was purchased from Goryo Chemicals (Japan). Kadcyla was purchased from Chugai Pharmaceutical Co., Ltd (Tokyo, Japan). Breast tumour cells were kindly provided by Dr Kurebayashi (Kawasaki Medical School). Nude mice (five-week-old female BALB/c nu/nu mice) were purchased from Nihon SLC Inc (Japan).

Absorption spectra were recorded with a spectrophotometer (V-670, Jasco). Fluorescence spectra were recorded with a spectrofluorometer (FP-8200, Jasco). Cellular imaging was performed with a fluorescence microscope (BZ-X700, Keyence). Flow cytometric analysis was performed using MACSQuant analyzer (Miltenyi Biotec Inc.) *In vivo* NIR fluorescence imaging was performed using a fluorescence system (Bruker MS-FX PRO).

### Preparation of recombinant proteins (Annexin V, EGFP–Annexin V and mPlum–Annexin V)

#### (a) Construction of the recombinant plasmids, pRSET–Annexin V, pRSET–EGFP–Annexin V, and pRSET–mPlum–Annexin V

Annexin V sequence was amplified by PCR from pET12a-PAPI, which was a gift from Jonathan Tait (Addgene plasmid #19961, B. L. Wood, D. F. Gibson, and J. F. Tait, Blood 1996, 88, 1873–1880). EGFP sequence was amplified by PCR from pEGFP-C1 plasmid (Clontech). mPlum sequence was amplified by PCR from pmPlum vector (Clontech). The PCR fragments were fused with pRSET plasmid (ThermoFisher) by using an InFusion HD cloning kit (Clontech).

#### (b) Expression of the recombinant proteins in *E. coli*

The pRSET–Annexin V, pRSET–EGFP–Annexin V and pRSET–mPlum–Annexin V plasmids were transformed into *E. coli* KRX competent cells (Promega). Transformed *E. coli* were grown as a preculture (2 mL) in LB medium containing ampicillin (100 μg mL^−1^) at 37 °C overnight. For large-scale cultures, the overnight culture (2 mL) was grown in 200 mL of LB medium containing ampicillin (100 μg mL^−1^) at 37 °C, until they approached to 0.6 of OD 600 (absorbance). To induce production of the targeted protein, isopropyl β-d-1-thiogalactopyranoside (0.2 mM) and l-rhamnose (0.1%) were added to the LB medium, and then incubated with shaking gently for 16 h at 18 °C.

#### (c) Extraction and purification of the recombinant proteins

The cells were harvested by centrifugation at 5000 × *g* for 10 min. The pellet was resuspended in 5 mL of binding buffer (50 mM Tris–HCl, 500 mM NaCl, 20 mM imidazole, pH 8.0). Before cell lysis, complete EDTA-free protease inhibitor cocktail tablets (1×, Roche) were added as a protease inhibitor. The suspension was sonicated on ice. Bursts of 10 seconds with intermediate intensity are repeated 7–10 times with a 10 second cooling period between each burst. The lysate was clarified by centrifugation at 20 000 × *g* for 30 min to eliminate cell debris. The supernatant was then purified by Ni Sepharose 6 Fast Flow (GE Healthcare). 0.5 mL of Sepharose media equilibrated with binding buffer was added to each 5 mL of lysed sample, and incubated with gentle agitation at 4 °C for 60 min. After the solution was transferred to an empty column, it was washed with binding buffer five column volumes. Lastly, the recombinant proteins were drained from the column by the addition of elution buffer (50 mM Tris–HCl, 500 mM NaCl, 500 mM imidazole, pH 8.0). The eluted fractions were further purified by a size-exclusion column (PD-10, GE Healthcare) to exchange buffer.

### ICG labelling of Kadcyla and recombinant proteins

ICG-NHS ester (1 mg) was resolved to 1 mL of anhydrous dimethyl sulfoxide. To an aqueous solution (0.01 M Na_2_CO_3_) of Kadcyla (1 mg mL^−1^) or recombinant proteins (1 mg mL^−1^), 30 μL of a dimethyl sulfoxide solution of ICG-NHS was dropwisely added. The coupling reaction was performed for 30 min at room temperature. The ICG conjugates were purified by a size-exclusion column (PD-10, GE Healthcare) to remove unreacted dyes.

### SDS PAGE

The proteins were run on a 5–20% polyacrylamide gel (Extra PAGE one Precast gel, Nacalai Tesque) in Tris–glycine–SDS buffer, 200 V for 40 min and stained with Coomassie Brilliant Blue (CBB Stain One Super, Nacalai Tesque). The sizes of Annexin V, EGFP–Annexin V and mPlum–Annexin V calculated from the amino acid sequence were 39.8 kDa, 66.7 kDa and 65.5 kDa, respectively. Precision Plus Protein Standard (BIO-RAD) was used as a size marker.

### Cellular imaging and flow cytometric analysis

KPL-4 cells were plated to 35 mm cell culture dishes (3 × 10^5^ cells per dish) and incubated in Dulbecco's Modified Essential Medium supplemented with 10% fetal bovine serum, 100 μg mL^−1^ penicillin, and 10 μg mL^−1^ streptomycin in 5% CO_2_ at 37 °C for overnight. Then, KPL-4 was exposed to Kadcyla (10 nM or none) for 72 h. All the cells floating in the medium and the cells that had detached during the PBS washing were collected, and the cells attached to the dish were carefully detached by trypsinization and added to the cell suspension. The cells were washed once with PBS and then resuspended in 1 mL of Annexin V binding buffer (Nacalai Tesque).

The cell suspension was divided into 100 μL aliquots, added with FITC–Annexin V (5 μl; FITC–Annexin V Apoptosis Detection Kit, Nacalai Tesque), ICG–EGFP–Annexin V (final concentration, 0.7 μM) or ICG–mPlum–Annexin V (final concentration, 0.7 μM) and incubated at room temperature for 15 minutes in the dark. As a blocking control, cell suspensions were preincubated with unlabeled recombinant Annexin V (final concentration 4 μM) for 15 minutes at room temperature prior to this staining step. Then, 400 μL of binding buffer was added to the stained cell suspension, which was passed through a 35 μm cell strainer before use in the next experiment. Quantification by flow cytometry was performed using the MACSQuant Analyzer (Miltenyi Biotec Inc.). FITC–Annexin V was collected through a FL2 filter (ex: 488 nm, em: 525 ± 25 nm). Fluorescence images were acquired with a fluorescence microscope (BZ-X700, Keyence Corp., Japan). The filter set for FITC and EGFP was ex: 470 ± 20 nm, em: 525 ± 25 nm. The filter set for mPlum was ex: 560 ± 20 nm, em: 590 nm LP. The filter set for ICG was ex: 769 ± 20 nm, em: 832 ± 19 nm.

### Preparation of tumour-bearing mice

A suspension of KPL-4 cells (∼10^7^ cells per mouse) was transplanted to the ventral side of 5 week old female BALB/c nu/nu mice. After several weeks, we selected a mouse bearing a tumour less than 10 mm in diameter for imaging.

### *In vivo* fluorescence imaging of tumour-bearing mice

For the fluorescence imaging of a breast-tumour, an aqueous solution (200 μL) of ICG-labelled Kadcyla (1 mg mL^−1^) was intravenously injected *via* a tail vein of the mouse. NIR fluorescence images of the tumour were taken 0, 1, 3, and 5 days after the injection of ICG labelled Kadcyla. Five days after of the probe, *ex vivo* images of a breast tumour and organs were taken.

For the fluorescence imaging of tumour-apoptosis, Kadcyla (200 μL, 1 mg mL^−1^) was intravenously injected *via* a tail vein of breast tumour-bearing mice. Three days after the injection of Kadcyla, ICG–EGFP (or Plum)–Annexin V or ICG–Annexin V (200 μL, 1 mg mL^−1^) was injected to the mice. Three days after the probes, fluorescence images of a mouse treated were taken.

Fluorescence images were taken using an *in vivo* fluorescence imaging system (Bruker, MS FX PRO). NIR fluorescence of ICG was observed at 830 ± 20 nm by excitation at 760 nm. Exposure time of the excitation light was 30 s. VIS fluorescence of EGFP and mPlum was observed at 515 ± 20 nm (ex: 470 nm) and 670 ± 20 nm (ex: 590 nm), respectively. Exposure time of the excitation light was 1 s for EGFP emission and 10 s for mPlum emission. Excitation light (400 W Xenon lamp) power was 30 μW cm^−2^ at a ventral side of a mouse.

## Ethical statement

Mice maintenance and animal experiments were performed in accordance with the Guidelines for Care and Use of Laboratory Animals of RIKEN and approved by the Animal Ethics Committee of RIKEN.

## Conflicts of interest

There are no conflicts to declare.

## Supplementary Material

RA-010-D0RA06495E-s001
