# Supplementary material for: Dual-colour (near-infrared/visible) emitting annexin V for fluorescence imaging of tumour cell apoptosis in vitro and in vivo
Source: RSC Adv. 2020 Oct 16;10(63):38244–50. doi: 10.1039/d0ra06495e (PMC9057337; doi:10.1039/d0ra06495e)
Supplement: RA-010-D0RA06495E-s001 [file RA-010-D0RA06495E-s001.pdf]

Electronic Supplementary Material (ESI) for RSC Advances.
This journal is © The Royal Society of Chemistry 2020

Electronic Supplementary Information

Dual-colour (near-infrared/visible) emitting annexin V for fluorescence imaging of tumour cell apoptosis *in vitro* and *in vivo*

Setsuko Tsuboi and Takashi Jin

RIKEN Center for Biosystems Dynamic Research (BDR), Furuedai 6-2-3, Suita, Osaka 565-0874, Japan. E-mail: tjin@riken.jp

Supplementary data

Fig. S1
Structure of pRSET-Annexin V, pRSET-EGFP-Annexin V, pRSET-mPlum-Annexin V plasmids.

Fig. S2
SDS PAGE of Annexin V, ICG-Annexin V, ICG-EGFP-Annexin V and ICG-mPlum-Annexin V.

Fig. S3
Absorption spectra of EGFP and mPlum before and after labelling with ICG-NHS.

Fig. S4
Changes in the fluorescence intensities of ICG, EGFP and mPlum emissions.

Fig. S5
Flow cytometric analysis and microscopy imaging of KPL-4 cells treated with Kadcyla.

Fig. S6
Blocking experiments for apoptosis-induced KPL-4 cells.

Fig. S7
Bright field (BF) and NIR fluorescence images of breast tumour-bearing mice injected with ICG-EGFP-Annexin V.

Fig. S8
Bright filed (BF) and VIS/NIR dual-colour fluorescence images of breast tumour-bearing mice.

Fig. S9
Bright field (BF) and NIR fluorescence images of breast tumour-bearing mice injected with ICG-Annexin V.

Fig. S10
NIR fluorescence image of breast tumour-bearing mice injected with ICG-EGFP and ICG-mPlum.

Table S1
Optical properties of ICG-Annexin V, ICG-EGFP-Annexin V, and ICG-mPlum-Annexin V.

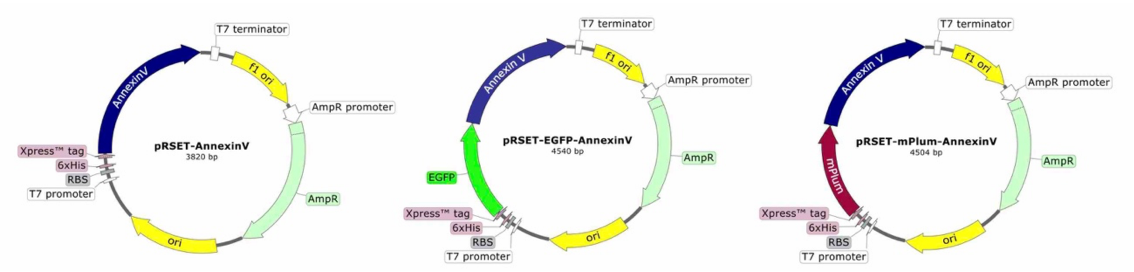

Fig. S1 Structure of pRSET-Annexin V, pRSET-EGFP-Annexin V, and pRSET-mPlum-Annexin V plasmids.

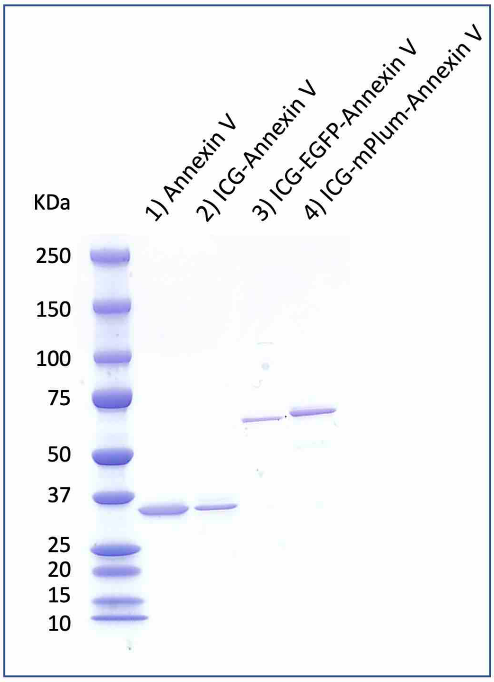

Fig. S2 SDS PAGE of annexin V, ICG-Annexin V, ICG-EGFP-Annexin V and ICG-mPlum-Annexin V.

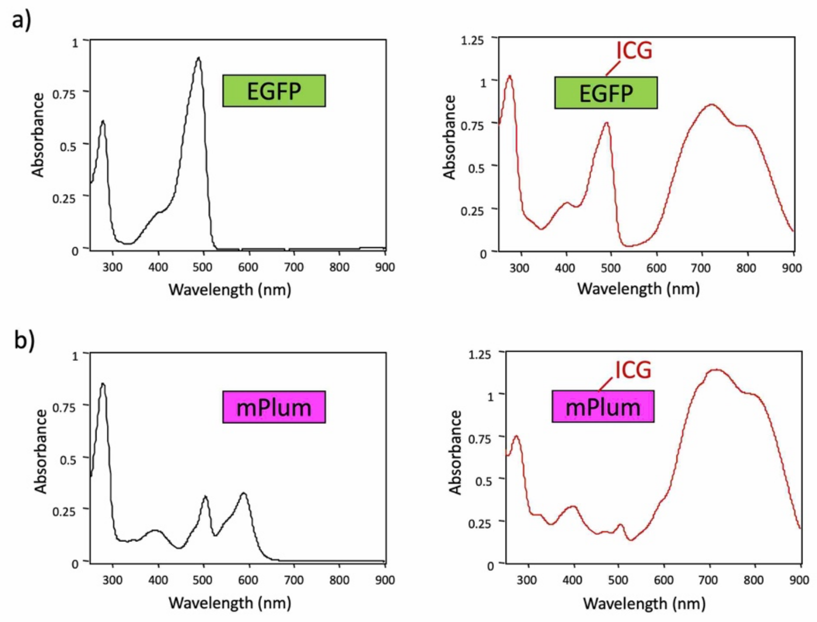

Fig. S3 Absorption spectra of EGFP and mPlum before and after labelling with ICG-NHS.

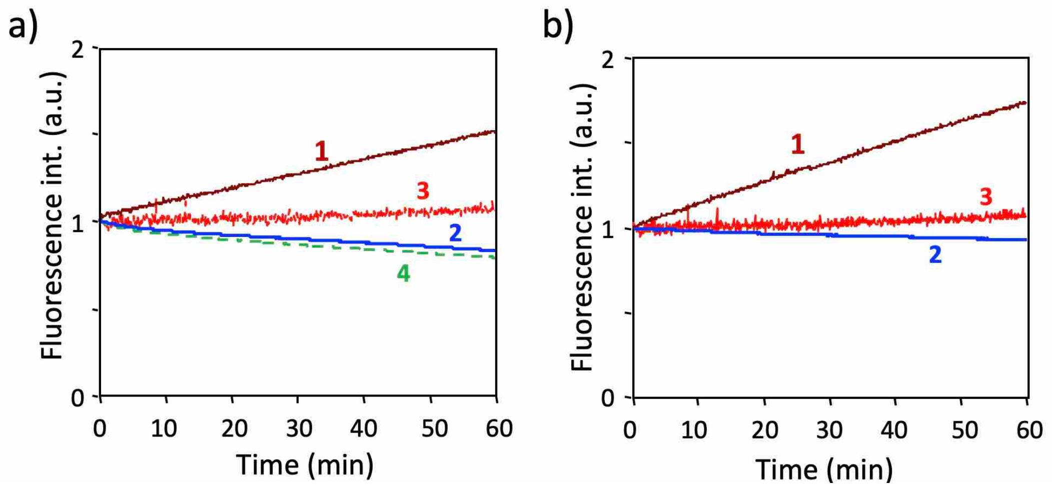

Fig. S4 Changes in the fluorescence intensities of ICG, EGFP and mPlum emissions in ICG-EGFP-Annexin V and ICG-mPlum-Annexin V by irradiation of excitation lights at 480 nm, 550 nm, and 780 nm (3 mW/cm^2): a) ICG-EGFP-Annexin V, 1: ICG emission (ex:780 nm, em 830 nm), 2: EGFP emission (ex:480 nm, em:515 nm); b) ICG-mPlum-Annexin V, 1: ICG emission (ex:780 nm, em: 830 nm), 2: mPlum emission (ex:550 nm, em: 670 nm). For comparison, fluorescence stability of ICG emission (3: red line, ex:780 nm, em 830 nm) in ICG-Annexin V and FITC emission (4: green line, ex:480 nm, em:515 nm) in FITC-Annexin V was also examined. The concentration of probes was 0.75 μM.

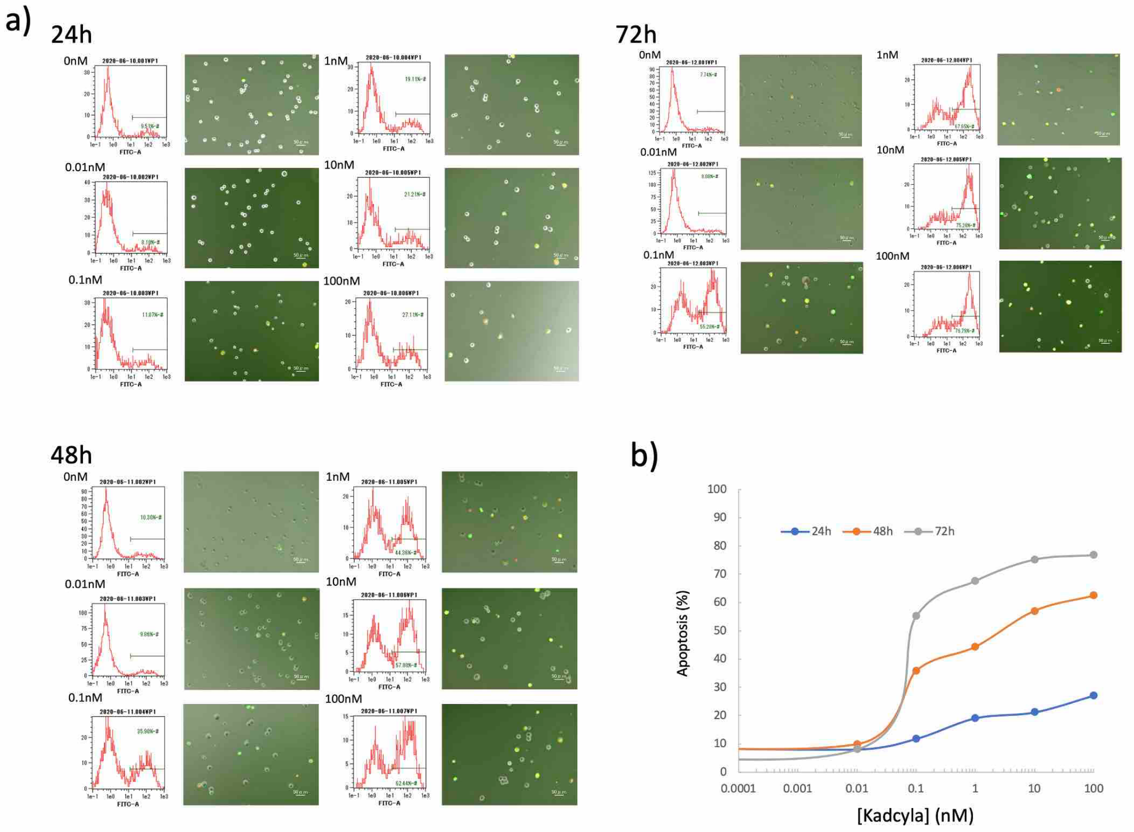

Fig. S5 a) Flow cytometric analysis and microscopy imaging of KPL-4 cells treated with Kadcyla (0-100 nM) for 24, 48, and 72h. Microscopy images merged a bright filed and fluorescence image. Green and red colours show the fluorescence from FITC-Annexin V and propidium iodide (PI), respectively. b) The graph shows the apoptosis (%) of KPL-cells treated with Kadcyla.

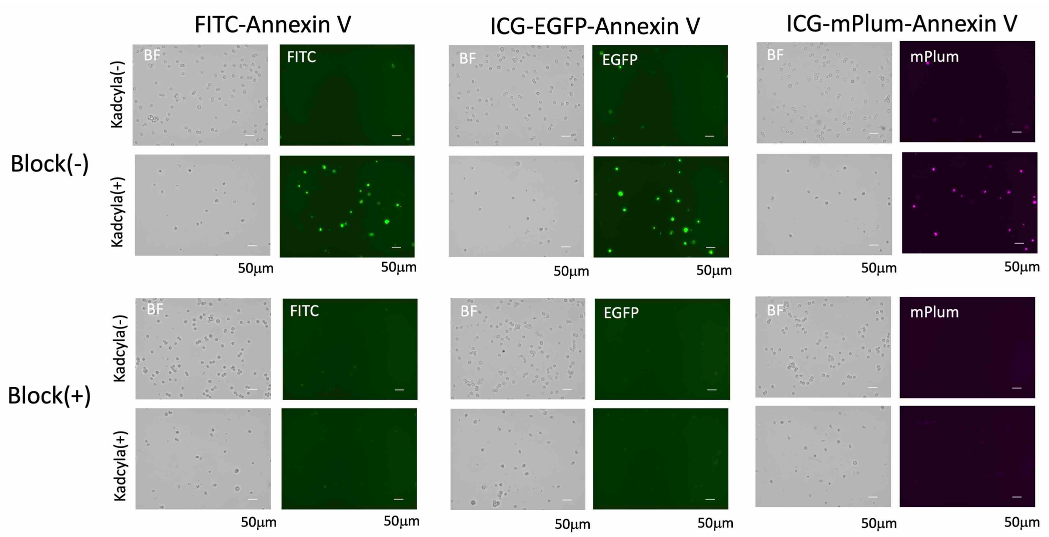

Fig. S6 Blocking experiments using unlabelled annexin V for apoptosis-induced KPL-4 cells. Cellular imaging was performed three days after the treatment of Kadcyla. Cells were stained with FITC-Annexin V (5 μL ; FITC-Annexin V Apoptosis Detection Kit, Nacalai Tesque), ICG-EGFP-Annexin V (0.7 μM) or ICG-mPlum-Annexin V (0.7 μM). As a blocking control, cell suspensions were preincubated with unlabeled recombinant annexin V (final concentration 4 μM) for 15 minutes prior to staining with the fluorescent probes.

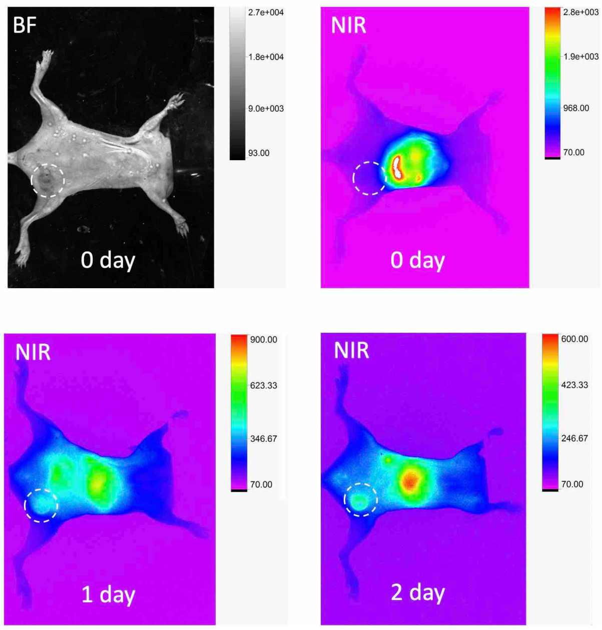

Fig. S7 Bright field and NIR fluorescence images of breast tumour-bearing mice treated with Kadcyla (0.2 mg). Two hundred μL of Kadcyla (1mg/mL) was intravenously injected *via* a tail vein of the mouse. ICG-EGFP-Annexin V was injected to the mouse three days after the injection of Kadcyla. NIR fluorescence images (em: 830 ± 20 nm) were taken 0, 1, an 2 days after the injection of ICG-EGFP-Annexin V. The dotted circles show the position of breast tumours in the mice.

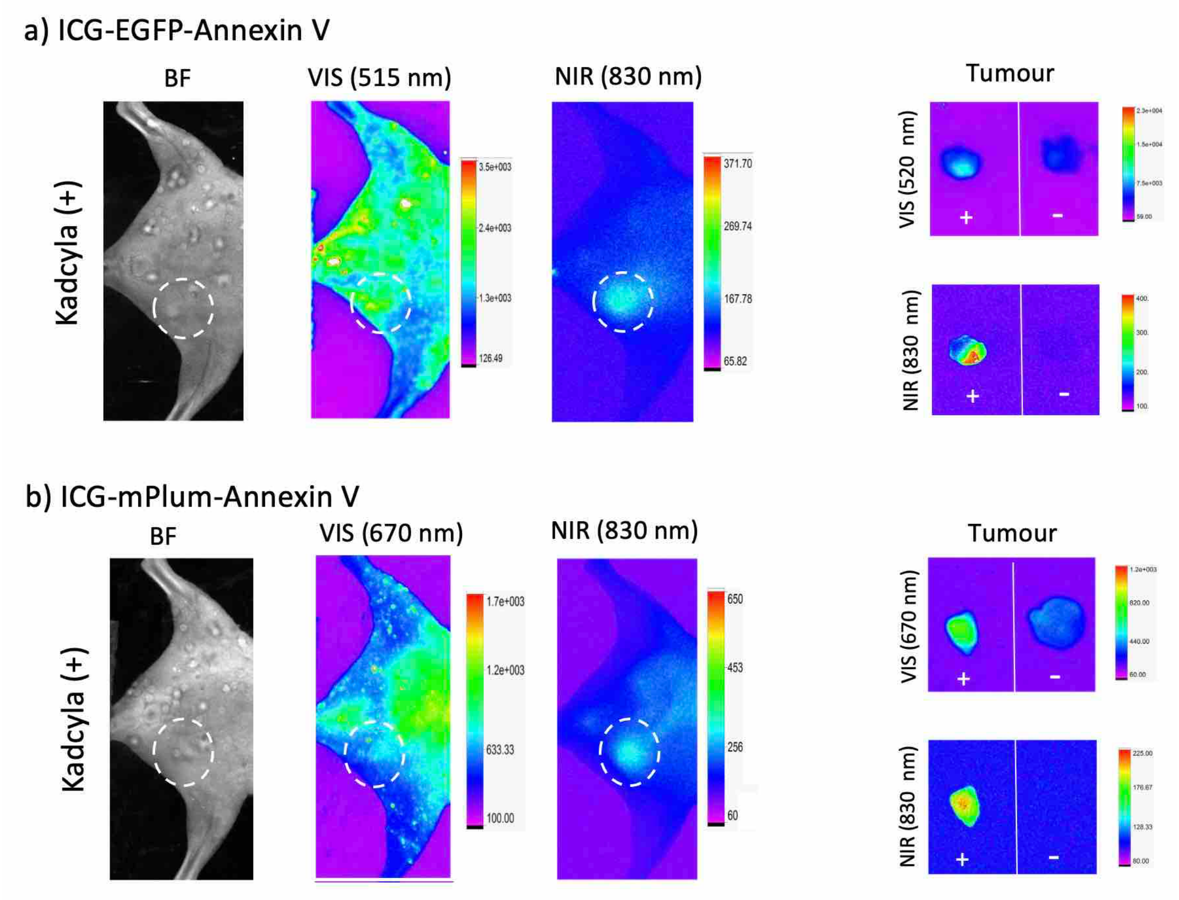

Fig. S8 Bright filed (BF) and VIS/NIR dual-colour fluorescence images of breast tumour-bearing mice: a) ICG-EGFP-Annexin V injected mouse and b) ICG-mPlum-Annexin V injected mouse. VIS fluorescence images were taken at 515 ± 20 nm for EGFP emission and 670 ± 20 nm for mPlum emission. NIR fluorescence images were taken at 830 ± 20 nm. Tumour apoptosis was induced by the injection of 200 μL of Kadcyla (1mg/mL). Three days after the injection of Kadcyla, ICG-EGFP-Annexin V (or ICG-mPlum-Annexin V) was injected to the Kadcyla treated mouse. Images were taken three days after the injection of the apoptosis probe. The dotted circles show the position of breast tumours in the mice. *Ex vivo* images show VIS and NIR fluorescence images of isolated the breast tumours. Control images (-) were taken for the tumours injected by no Kadcyla and probes.

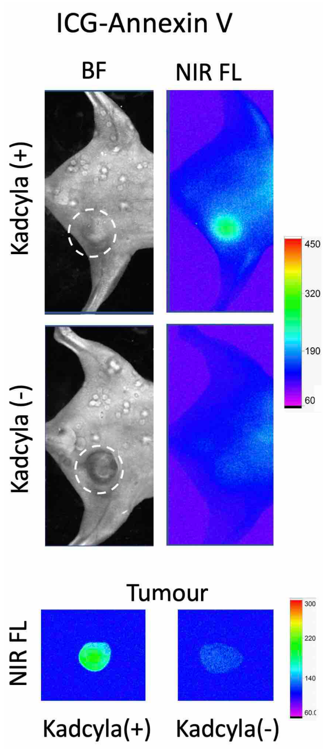

Fig. S9 Bright field (BF) and NIR fluorescence images of breast tumour-bearing mice treated with and without Kadcyla. Two hundred μL of Kadcyla (1mg/mL) was intravenously injected *via* a tail vein of the mouse. ICG-Annexin V was injected to the mouse three days after the injection of Kadcyla. NIR fluorescence images (em: 830 ± 20 nm) were taken three days after the injection of ICG-Annexin V. The dotted circles show the position of breast tumours in the mice. The *ex vivo* images show the NIR fluorescence images of isolated the breast tumours.

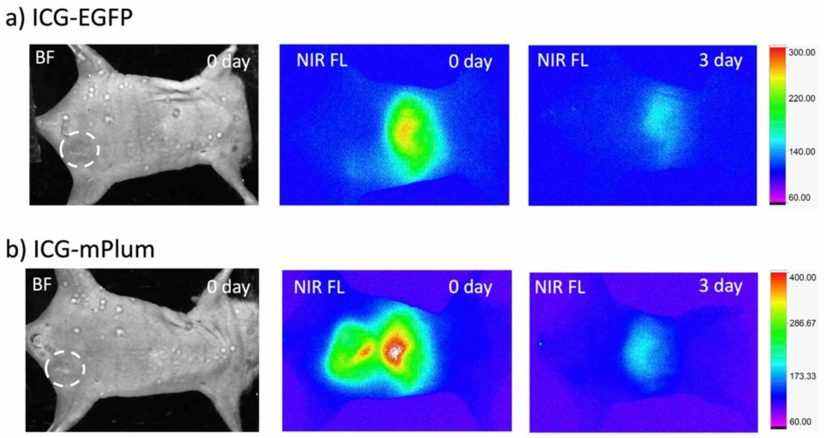

Fig. S10 NIR fluorescence image of breast tumour-bearing mice injected with ICG-EGFP (200 μL, 1 mg/mL) or ICG-mPlum (200 μL, 1mg/mL). Fluorescent probes were injected to the mice *via* their tail veins. NIR fluorescence was observed at 830 ± 20 nm. The dotted circles show the positions of breast tumours in the mice.

Table S1 Optical properties of ICG-Annexin V, ICG-EGFP-Annexin V, and ICG-mPlum-Annexin V.

	M. W.*	ε (280 nm)	Ex_{max} / Em_{max} (nm)	QY (%)
ICG-Annexin V	41.4 kDa	31,000	800/820 (ICG)	~1
ICG-EGFP-Annexin V	68.3 kDa	42,000	490/515 (EGFP)	35
			800/820 (ICG)	~ 1
ICG-mPlum-Annexin V	67.1 kDa	59,000	590/640 (mPlum)	7
			800/820 (ICG)	~ 1

* Calculated by assuming that the labeling ratio of ICG per protein is 2.
